# Staphylococcus aureus and Pseudomonas aeruginosa Isolates from the Same Cystic Fibrosis Respiratory Sample Coexist in Coculture

**DOI:** 10.1128/spectrum.00976-22

**Published:** 2022-07-18

**Authors:** Eryn E. Bernardy, Vishnu Raghuram, Joanna B. Goldberg

**Affiliations:** a Department of Biology, Elon University, Elon, North Carolina, USA; b Division of Pulmonary, Asthma, Cystic Fibrosis, and Sleep, Department of Pediatrics, Emory University School of Medicine, Atlanta, Georgia, USA; c Microbiology and Molecular Genetics Program, Graduate Division of Biological and Biomedical Sciences, Laney Graduate School, Emory University, Atlanta, Georgia, USA; University of Maryland School of Pharmacy

**Keywords:** *Pseudomonas aeruginosa*, *Staphylococcus aureus*, cystic fibrosis, coinfection, lung disease

## Abstract

Respiratory infections with bacterial pathogens remain the major cause of morbidity in individuals with the genetic disease cystic fibrosis (CF). Some studies have shown that CF patients that harbor both Staphylococcus aureus and Pseudomonas aeruginosa in their lungs are at even greater risk for more severe and complicated respiratory infections and earlier death. However, the drivers for this worse clinical condition are not well understood. To investigate the interactions between these two microbes that might be responsible for their increased pathogenic potential, we obtained 28 pairs of S. aureus and P. aeruginosa from the same respiratory samples from 18 individuals with CF. We compared the survival of each S. aureus CF isolate cocultured with its corresponding coinfecting CF P. aeruginosa to when it was cocultured with non-CF laboratory strains of P. aeruginosa. We found that the S. aureus survival was significantly higher in the presence of the coinfecting P. aeruginosa compared to laboratory P. aeruginosa strains, regardless of whether the coinfecting isolate was mucoid or nonmucoid. We also tested how a non-CF S. aureus strain, JE2, behaved with each P. aeruginosa CF isolate and found that its interaction was similar to how the CF S. aureus isolate interacted with its coinfecting P. aeruginosa. Altogether, our work suggests that interactions between S. aureus and P. aeruginosa that promote coexistence in the CF lung are isolate-dependent and that this interaction appears to be driven mainly by P. aeruginosa.

**IMPORTANCE** Previous studies have shown that in laboratory settings, Pseudomonas aeruginosa generally kills Staphylococcus aureus. However, these bacteria are often found coinfecting the lungs of cystic fibrosis (CF) patients, which has been associated with worse patient outcomes. To investigate the interactions between these two bacteria, we competed 28 coinfection pairs obtained from the same lung samples of 18 different CF patients. We compared these results to those we previously reported of each CF S. aureus isolate against a non-CF laboratory strain of P. aeruginosa. We found that S. aureus survival against its corresponding coinfection P. aeruginosa was higher than its survival against the laboratory strain of P. aeruginosa. These results suggest that there may be selection for coexistence of these microbes in the CF lung environment. Further understanding of the interactions between P. aeruginosa and S. aureus will provide insights into the drivers of coexistence and their impact on the host.

## INTRODUCTION

The majority of the mortality in the inherited disease cystic fibrosis (CF) is due to bacterial lung infections. It is now appreciated that these respiratory infections are polymicrobial. The most common pathogens identified by culture methods include Pseudomonas aeruginosa, Staphylococcus aureus, Haemophilus influenzae, Stenotrophomonas maltophilia, *Achromobacter* species, and the Burkholderia cepacia complex. Of these, S. aureus has taken over as the microbe most commonly isolated, while P. aeruginosa remains associated with the majority of the morbidity and mortality in people living with CF (1).

Studies from our group and others have shown that CF patients that have lung infections with both S. aureus and P. aeruginosa are at greater risk for more severe disease and complicated respiratory infections than those infected with either S. aureus or P. aeruginosa alone (2–4), while other studies have shown no difference in the clinical outcomes between CF patients infected with P. aeruginosa alone versus those coinfected with P. aeruginosa and S. aureus (2, 5, 6). Differences in the patient cohorts as well as the nature of the isolates themselves have been suggested as potential reasons for these disparate findings. However, it remains poorly understood how these species can coexist (i.e., survive together in the same environment) in the CF lung despite studies from our lab and many others showing that S. aureus is typically killed when cocultured with P. aeruginosa *in vitro* (7–10).

To begin to address this question, we examined a collection of S. aureus isolates from respiratory samples obtained from CF patients enrolled in the Emory Cystic Fibrosis Biospecimen Registry. We previously reported the outcomes of competition between these CF S. aureus isolates and isogenic nonmucoid and mucoid variants of the laboratory P. aeruginosa strain PAO1 using a coculture assay developed in our laboratory. We categorized these CF S. aureus isolates based on the competition outcomes: killed by nonmucoid PAO1 but not mucoid PAO1, killed by both, or killed by neither. However, it is not known how these CF S. aureus fare against P. aeruginosa isolates that were present in the same CF respiratory sample—here referred to as “coinfection pairs.”

In this study, we competed 28 coinfection pairs of S. aureus and P. aeruginosa against each other. These isolates were obtained from the respiratory samples of 18 CF patients. We also compared the survival of the coinfection pairs in competition against the previously reported outcomes of each CF S. aureus isolate against mucoid and nonmucoid PAO1. We found that S. aureus survival against its corresponding coinfection P. aeruginosa pair was higher than its survival against a non-CF laboratory P. aeruginosa. This was true regardless of the P. aeruginosa mucoid status, suggesting possible adaptation between these microbes in the CF lung environment. Moreover, we found that survival of non-CF S. aureus strain JE2 was comparable to that of CF S. aureus when competed against CF P. aeruginosa. This suggests that P. aeruginosa primarily drives the coexistence of these two microbes. These findings set the stage for future studies that will dissect the mechanisms that allow both microbes to survive together in the CF lung.

## RESULTS

### S. aureus survives better with its coinfecting CF P. aeruginosa.

To determine the interaction between coinfection pairs, we performed coculture experiments on S. aureus isolates with P. aeruginosa isolates that were obtained from the same respiratory sample. We calculated the CFU/mL fold change for S. aureus grown in the presence of its coinfecting P. aeruginosa isolate compared to S. aureus in monoculture (Table 1). We then compared these data to what we had previously obtained for these same S. aureus isolates in the presence of P. aeruginosa strain PAO1 (10). Since our previous studies had determined that S. aureus survived better in the presence of mucoid P. aeruginosa than nonmucoid P. aeruginosa (10), we separated our analysis depending on whether the coinfecting P. aeruginosa isolate was mucoid or nonmucoid.

**TABLE 1:** Survival of S. aureus isolates when cocultured with concurrently isolated P. aeruginosa, grouped by patient ID

Patient information		S. aureus	P. aeruginosa		CFU/mL fold change of S. aureus with P. aeruginosa^*a*^
Patient ID	Date of collection (mo/day/yr)	Isolate name	Isolate name	Mucoidy	
102	4/24/2012	Sa_CFBR_17	CFBR102_Pae_20120424_S_Pa38	Mucoid	7.47E-01
105	10/25/2011	Sa_CFBR_29	CFBR105_Pae_20111025_S_EBPa06	Mucoid	9.08E-01
			CFBR105_Pae_20111025_S_EBPa07	Mucoid	9.13E-01
	1/17/2012	Sa_CFBR_30	CFBR105_Pae_20120117_S_EBPa09	Mucoid	7.50E-01
	4/16/2012	Sa_CFBR_31	CFBR105_Pae_20120416_S_EBPa11	Mucoid	7.93E-01
	6/27/2012	Sa_CFBR_32	CFBR105_Pae_20120627_S_EBPa13	Mucoid	9.16E-01
	8/2/2012	Sa_CFBR_33	CFBR105_Pae_20120802_S_EBPa15	Mucoid	7.45E-01
120	6/27/2012	Sa_CFBR_18	CFBR120_Pae_20120627_S_Pa41	Nonmucoid	1.01E + 00
123	2/22/2012	Sa_CFBR_19	CFBR123_Pae_20120222_S_Pa44	Nonmucoid	9.74E-03
			CFBR123_Pae_20120222_S_Pa43	Mucoid	3.61E-01
134	3/26/2012	Sa_CFBR_10	CFBR134_Pae_20120326_S_Pa20	Nonmucoid	5.13E-01
			CFBR134_Pae_20120326_S_Pa19	Mucoid	1.16E + 00
149	6/27/2012	Sa_CFBR_20	CFBR149_Pae_20120627_S_Pa45	Mucoid	5.97E-01
152	1/25/2012	Sa_CFBR_06	CFBR152_Pae_20120125_S_Pa14	Mucoid	3.27E-01
170	2/1/2012	Sa_CFBR_07	CFBR170_Pae_20120201_S_Pa15	Mucoid	1.04E + 00
171	2/8/2012	Sa_CFBR_23	CFBR171_Pae_20120208_S_Pa84	Nonmucoid	1.08E + 00
196	2/21/2012	Sa_CFBR_08	CFBR196_Pae_20120221_S_Pa17	Mucoid	9.64E-01
201	1/17/2012	Sa_CFBR_24	CFBR201_Pae_20120117_S_Pa80	Nonmucoid	4.15E-01
			CFBR201_Pae_20120117_S_Pa81	Nonmucoid	6.15E-01
			CFBR201_Pae_20120117_S_Pa82	Mucoid	5.04E-01
219	5/29/2012	Sa_CFBR_09	CFBR219_Pae_20120529_S_Pa18	Mucoid	6.47E-01
309	5/10/2017	Sa_CFBR_37	CFBR309_Pae_20170510_S_EBPa20	Nonmucoid	3.66E-03
336	4/5/2017	SA_CFBR_08	CFBR336_Pae_20170405_S_EBPa24	Mucoid	2.54E-01
447	4/5/2017	Sa_CFBR_43	CFBR447_Pae_20170405_S_EBPa28	Mucoid	1.55E-04
509	5/25/2017	Sa_CFBR_46	CFBR509_Pae_20170525_S_EBPa32	Nonmucoid	2.85E-04
515	2/17/2017	Sa_CFBR_47	CFBR515_Pae_20170217_S_EBPa34	Nonmucoid	1.47E + 00
530	4/5/2017	Sa_CFBR_48	CFBR530_Pae_20170405_S_EBPa36	Nonmucoid	1.13E + 00
			CFBR530_Pae_20170405_S_EBPa37	Mucoid	2.02E + 00

a The fold change was calculated as described in Materials and Methods.

**FIG 1:**
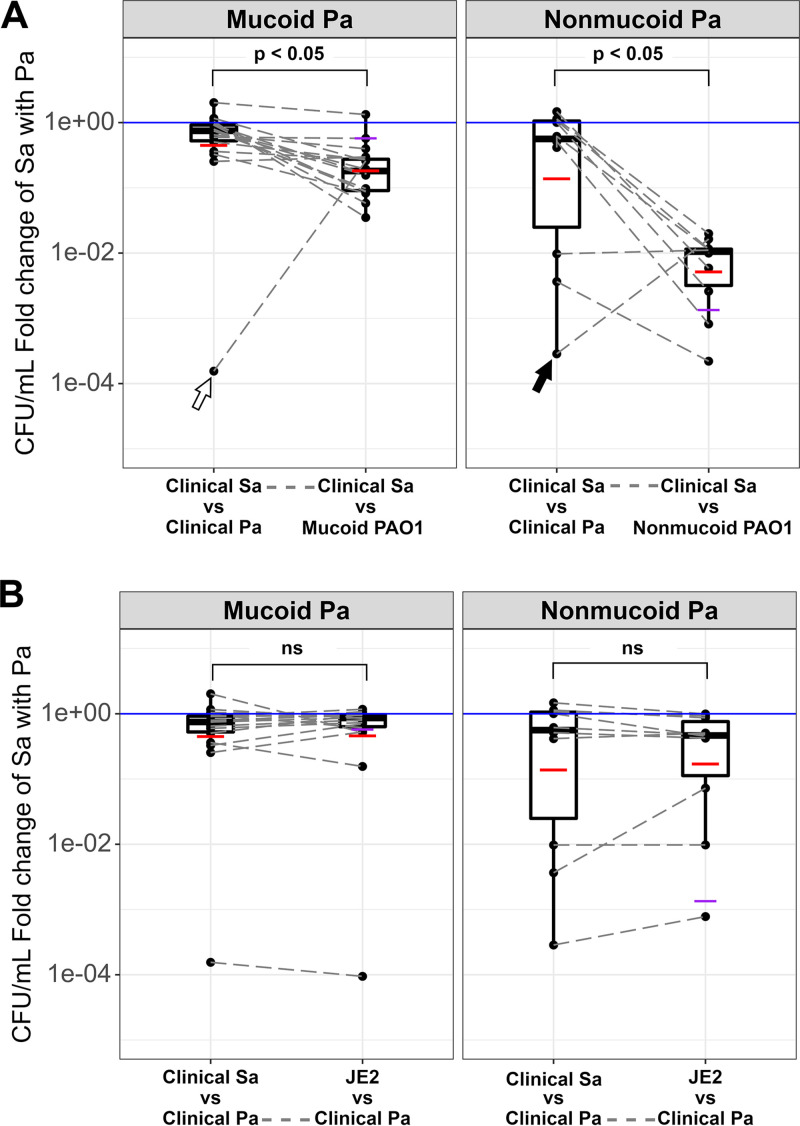
S. aureus (Sa) survives better with its coinfecting cystic fibrosis (CF) P. aeruginosa (Pa). The CFU/mL fold change of S. aureus when cocultured with P. aeruginosa was determined as described in Materials and Methods. The purple horizontal line shows the CFU/mL fold change of the reference S. aureus strain JE2 when cocultured with mucoid P. aeruginosa PAO1 (left panels in A and B) or nonmucoid P. aeruginosa PAO1 (right panels in A and B). The black horizontal lines inside the boxplots show the median, and the red horizontal lines show the mean. The white boxes represent the interquartile range (IQR), and the whiskers represent values up to 1.5 times the first or third quartile. The blue solid line shows a fold change of 1, suggesting no change when grown with P. aeruginosa compared to monoculture. (A) Boxplot of CFU/mL fold change of CF S. aureus cocultured with its concurrently isolated CF P. aeruginosa or mucoid/nonmucoid PAO1. Dots represent the average CFU/mL fold change of each S. aureus isolate, and the gray dashed lines connect dots that correspond to the same S. aureus isolate. The Wilcoxon signed rank test showed a significant difference between the mean CFU/mL fold change of CF S. aureus when cocultured with CF P. aeruginosa compared to the mean CFU/mL fold change of CF S. aureus when cocultured with mucoid (*P* = 5.089e-11, Shapiro-Wilk *P* = 0.001) or nonmucoid PAO1 (*P* = 1.847e-05, Shapiro-Wilk *P* = 3.648e-05). Arrows represent outliers, as described in text. (B) Boxplot of the CFU/mL fold change of CF S. aureus or reference strain JE2 cocultured with its concurrently isolated CF mucoid/nonmucoid P. aeruginosa. Dots represent the average CFU/mL fold change of each S. aureus isolate, and the gray dashed lines connect dots that correspond to the same P. aeruginosa isolate. The Wilcoxon signed rank test/Welch’s *t* test showed no significant difference between the mean CFU/mL fold change of CF S. aureus when cocultured with CF P. aeruginosa compared to the mean CFU/mL fold change of reference strain JE2 when cocultured with CF P. aeruginosa (*P* = 0.26/0.25, for mucoid/nonmucoid, respectively, Shapiro-Wilk *P* = 0.013/0.078; ns, not significant). The average fold change was calculated from at least three biological replicates (see Table S1 for raw data).

We compared the CFU/mL fold change of CF S. aureus cocultured with their mucoid coinfection partner P. aeruginosa (“Clinical Sa vs Clinical Pa”) to the CFU/mL fold change of the same CF S. aureus cocultured with the non-CF mucoid PAO1 (“Clinical Sa vs Mucoid PAO1”) (Fig. 1A, left “mucoid” panel; *P* = 5.089e-11). Similarly, we compared the CFU/mL fold change of CF S. aureus cocultured with their nonmucoid coinfection partner P. aeruginosa (“Clinical Sa vs Clinical Pa”) to the CFU/mL fold change of the same CF S. aureus cocultured with the non-CF nonmucoid PAO1 (“Clinical Sa vs nonmucoid PAO1”) (Fig. 1A, right “nonmucoid” panel; *P* = 1.847e-05). As seen in each panel in Fig. 1A, that the data show the “Clinical Sa vs Clinical Pa” survival was significantly higher than the “Clinical Sa vs mucoid/nonmucoid PAO1” survival, indicating that the CF S. aureus isolates survived better when cocultured with their coinfecting P. aeruginosa (overall *P* < 0.05).

To distinguish whether the increase in S. aureus survival was due to reduced killing by P. aeruginosa or increased resistance by S. aureus, we measured the survival of non-CF S. aureus JE2 against each CF P. aeruginosa isolate. We calculated the CFU/mL fold change of JE2 in coculture with CF P. aeruginosa, as described above. We then compared the survival of JE2 against CF P. aeruginosa with the survival of the coinfecting CF S. aureus against the same CF P. aeruginosa. We found no significant difference in the response shown by the CF and non-CF S. aureus to the CF P. aeruginosa. This was true regardless of whether the S. aureus strains were tested against mucoid or nonmucoid P. aeruginosa (Fig. 1B, *P* = 0.26 for “mucoid,” *P* = 0.25 for “nonmucoid”). These results suggested that the increased survival of CF S. aureus may be driven by reduced killing by P. aeruginosa, as the CF-adapted P. aeruginosa showed reduced killing of even a non-CF S. aureus strain.

Mucoid and nonmucoid P. aeruginosa isolates were collected concurrently from patients 123, 134, 201, and 530 (Table 1). Previous studies had noted that mucoid P. aeruginosa strains were more permissive than nonmucoid isolates to S. aureus (11). Interestingly, we only found this to be the case for P. aeruginosa isolates from patient 123: as expected the mucoid isolate from this patient was more permissive than the nonmucoid isolate when cocultured with the coinfecting S. aureus isolate. On the other hand, mucoid and nonmucoid isolates that were collected concurrently from patients 134, 201, and 530 seemed to show similar results to one another; all seemed to promote coexistence (Table 1).

We did observe a few outliers in Fig. 1A. In the left panel, the white arrow highlights the data related to patient 447: Sa_CFBR_43 vs CFBR447_Pae_20170405_EBPa28. In the right panel, the black arrow highlights the data related to patient 509: Sa_CFBR_46 vs CFBR509_Pae_20170525_EBPa32. Both these S. aureus isolates were killed more readily by their coinfecting pair partner. The two P. aeruginosa isolates were also able to readily kill the reference S. aureus strain JE2 (comparing Fig. 1A and B and Fig. S1 in the supplemental material). These isolates are being investigated further.

To determine whether P. aeruginosa and S. aureus coinfecting isolates were specifically coevolving together to promote coexistence, we performed coculture experiments with non-coinfecting isolates. We chose 3 P. aeruginosa isolates (2 nonmucoid and 1 mucoid) and cocultured them with 4 different S. aureus isolates from different patients and calculated the CFU/mL fold change of S. aureus. For these studies we did not choose any of the outlier P. aeruginosa or S. aureus isolates (Fig. 1A, white and black arrows). We found that the two nonmucoid strains (Fig. S2A and B) showed the same level of killing of the non-coinfecting S. aureus as they did with their coinfecting isolate. Interestingly, this was independent on whether the non-coinfecting S. aureus was killed by its own coinfection isolate. On the other hand, we noted that the mucoid P. aeruginosa isolate (Fig. S2C) was able to kill non-coinfecting S. aureus isolates, even though these S. aureus isolates coexisted with their respective coinfection isolates, as did the S. aureus isolate coinfecting with this mucoid P. aeruginosa. (Fig. S2, Table S3). This suggests that coexistence may also be affected by specific isolate-dependent interactions.

### P. aeruginosa survives similarly with its coinfecting CF S. aureus as it does with JE2.

**FIG 2:**
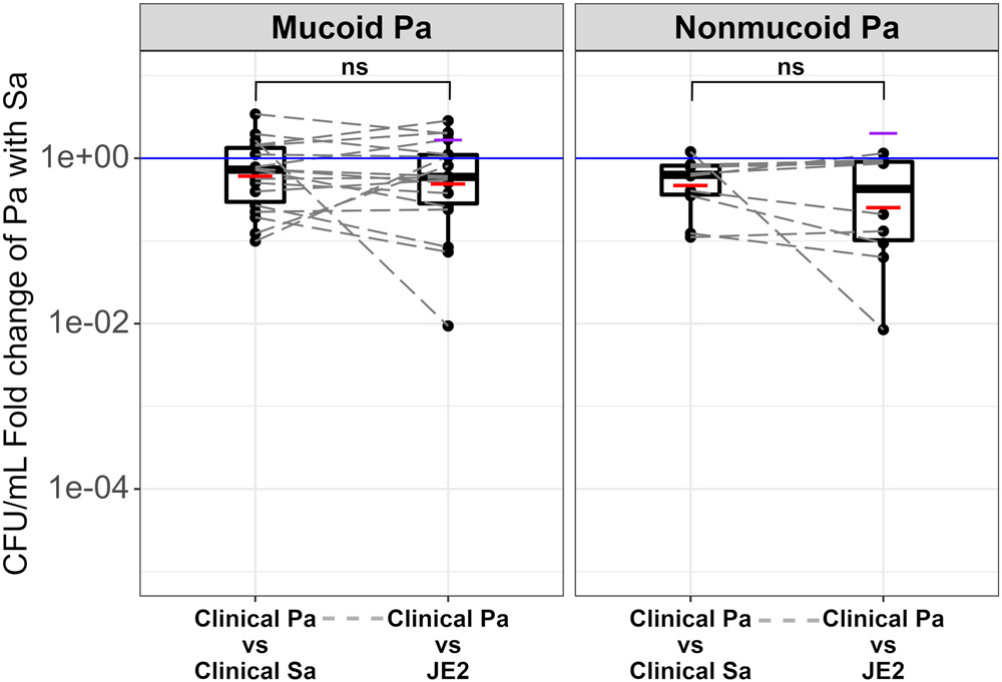
P. aeruginosa (Pa) survives similarly with its coinfecting cystic fibrosis (CF) S. aureus (Sa) and JE2. The CFU/mL fold change of P. aeruginosa when cocultured with S. aureus was determined as described in Materials and Methods. The purple horizontal line shows the CFU/mL fold change of mucoid P. aeruginosa PAO1 or nonmucoid PAO1 when cocultured with S. aureus JE2. The boxplots of the CFU/mL fold change of mucoid and nonmucoid CF P. aeruginosa cocultured with its concurrently isolated CF S. aureus or reference strain JE2 are shown. The black horizontal lines inside the boxplots show the median, and the red horizontal lines show the means. The white boxes represent the interquartile range (IQR), and the whiskers represent values up to 1.5 times the first or third quartile. The blue solid line shows a fold change of 1. Dots represent the average CFU/mL fold change of each P. aeruginosa isolate, and the gray dashed lines connect dots that correspond to the same P. aeruginosa isolate. The Wilcoxon signed rank test showed no significant difference between the mean CFU/mL fold change of CF P. aeruginosa when cocultured with its concurrently isolated CF S. aureus compared to the mean CFU/mL fold change of CF P. aeruginosa when cocultured with reference strain JE2 (*P* = 0.88/*P* = 0.19, for mucoid/nonmucoid, respectively. Shapiro-Wilk *P* = 4.533e-13/0.0001228; ns, not significant). The average fold change was calculated from at least three biological replicates (see Table S1 for raw data).

While P. aeruginosa has not been previously found to be negatively impacted by S. aureus, we also tested the survival of P. aeruginosa with its coinfecting S. aureus as well as with JE2 (Table S1). As seen in Fig. 2, most P. aeruginosa isolates survived similarly in the presence of their coinfecting S. aureus isolate compared to their survival in the presence of JE2. This happened regardless of whether the P. aeruginosa was mucoid (Fig. 2, left-hand panel; *P* = 0.88) or nonmucoid (Fig. 2, right-hand panel; *P* = 0.19). This indicated that there was little effect on survival of P. aeruginosa by coculture of the S. aureus under the conditions of this assay.

## DISCUSSION

Multiple studies have shown that CF patients coinfected with both S. aureus and P. aeruginosa are at greater risk for more severe and complicated respiratory infections (2–4, 6); however, the mechanisms responsible for these outcomes are not well understood. To uncover the reason for the worsening clinical manifestation, the processes allowing these two microbes to survive together need to be better understood. Various studies have shown that different stages of growth and environmental conditions, including media and planktonic versus biofilm modes of growth, can promote the coexistence of S. aureus and P. aeruginosa (9, 12, 13). In some other cases, it has been found that bacterial segregation promotes survival (13). On the other hand, many *in vitro* studies have shown that P. aeruginosa itself or P. aeruginosa factors, such as secreted LasA and rhamnolipids, can lyse or kill S. aureus (7, 14–17). We and others have previously observed that decreased expression of some of these factors in the context of mucoid conversion of P. aeruginosa promotes coexistence with S. aureus (11, 18). Some other studies have noted the physiological conditions that allow S. aureus and P. aeruginosa to survive and grow together (8, 19, 20). Many of the studies to uncover the mechanism of competition or coexistence have utilized laboratory isolates; however, more recently, investigations have been performed with S. aureus and P. aeruginosa clinical isolates (21–24).

Our goal here was to add to this growing list of studies by investigating pairs of clinical isolates of these bacteria obtained from the same patient sample on the same day. By studying paired, particularly longitudinal isolates we hoped to glean insights into novel mechanisms of interactions between these two pathogens. We examined 28 pairs of isolates obtained from 18 CF individuals; 5 of these people provided multiple samples longitudinally. We hypothesized that isolates of S. aureus would survive better with P. aeruginosa obtained concurrently compared to a typical P. aeruginosa laboratory strain, and any P. aeruginosa or S. aureus that behaved differently could be a source for future comparative studies to identify potential mechanisms of coexistence.

Overall, our data generally supported our hypothesis: we showed that CF S. aureus isolates survive better with their coinfecting P. aeruginosa isolates than with P. aeruginosa PAO1. We also separated our data based on the mucoid status of P. aeruginosa isolates in this study (mucoid or nonmucoid) since we know that this phenotype impacts the interaction with S. aureus (11). We noted that the difference in survival was more pronounced when comparing the interaction between S. aureus and the nonmucoid P. aeruginosa isolates versus S. aureus and the mucoid P. aeruginosa isolates (Fig. 1A). This suggests, as has been previously shown, that mucoidy itself is an adaptation that facilitates coexistence (11). We also observed no difference in the interaction of these coinfecting pairs in our longitudinal samples (all coexisted). Interestingly, when the S. aureus reference strain JE2 was cocultured with these P. aeruginosa CF isolates, it showed equivalent susceptibility to P. aeruginosa killing as the coinfecting S. aureus isolate (Fig. 1B). Thus, these results are not perfectly aligned with our original hypothesis, as the reference S. aureus strain was not from coinfection, which has led us to conclude that P. aeruginosa is the main driver of this coexistence, as has been suggested by previous studies from our lab and others (11, 25). Moreover, when we competed non-coinfecting CF isolates of P. aeruginosa and S. aureus, we found that S. aureus can either be killed by or coexist with P. aeruginosa regardless of whether or not the two isolates are coinfection pairs (Fig. S2). This suggests that coexistence is isolate-dependent, and while P. aeruginosa may be the main driver of coexistence, S. aureus also plays a role.

The two observed outlier S. aureus and P. aeruginosa coinfection pairs in Fig. 1A (white and black arrows) are currently being investigated. The fact that these two P. aeruginosa strains are able to kill both their corresponding coinfection S. aureus partner and JE2 supports the idea that P. aeruginosa drives the interaction. In addition, one of these outlier S. aureus isolates (SA_CFBR_43) may have P. aeruginosa strain PAO1-specific resistance mechanisms according to our previous study (10).

We are aware that our study has its limitations. While the S. aureus and P. aeruginosa were obtained from the same clinical sample, the interactions we are examining are all *in vitro*, and our assay, by design, promotes the interaction between these two different species. Also, we only examined individual isolates that had been retrieved by the clinical microbiology laboratory. We know that P. aeruginosa is phenotypically and genotypically heterogenous in this environment (26, 27), and some recent studies have also suggested that S. aureus may be similarly heterogenous (6, 24, 28, 29). Thus, the single isolates that we examined may only represent a subset of the genotypes/phenotypes present in the respiratory sample. Currently, we are obtaining panels and pools of isolates from clinical CF samples to determine the genotypic and phenotypic variability and their impact on coexistence. Thus, whether and how these genotypes/phenotypes correlate with the clinical status of a person with CF at the time the sample was collected will be an important area for future investigations.

It is also the case that S. aureus and P. aeruginosa are not the only inhabitants in the CF lung and that other microbes might impact the interactions of these two bacteria. However, even with these recognized shortcomings, our study supports the hypothesis that S. aureus and P. aeruginosa isolated from the same CF respiratory sample have adapted to promote their coexistence within the CF lung. And since coinfection is a more deadly situation for people living with CF, understanding what drives S. aureus*-*P. aeruginosa coexistence could allow us to devise ways of disrupting this interaction to improve patients’ prognosis.

## MATERIALS AND METHODS

### Bacterial strains.

All bacterial isolates used in this study were obtained from patients enrolled in the Emory Cystic Fibrosis Biospecimen Registry (CFBR) (Table 1). The S. aureus isolates have been previously described, sequenced (30), and characterized (10); their previous reported interaction with mucoid and nonmucoid P. aeruginosa PAO1 is included in Table S1. S. aureus JE2 is a USA300 derivative (31). P. aeruginosa isolates were obtained from the same clinical samples. The mucoid phenotype of P. aeruginosa was assessed by visualization after overnight growth on lysogeny broth (LB) agar and Pseudomonas isolation agar (PIA; BD Difco) at 37°C.

### Coculture assay.

We performed a quantitative coculture assay previously described in detail (10). We grew the isolates of interest overnight at 37°C in LB from single colonies taken from PIA for P. aeruginosa and Staphylococcus isolation agar (SIA; Trypticase soy agar [TSA] BD BBL with 7.5% NaCl) for S. aureus. These cultures were back-diluted to an optical density of 0.05 and mixed in a 1:1 ratio, or with sterile LB as monoculture controls; 10 μL of each mixture was placed on a 0.45-μm Millipore filter (Millipore-MM_NF-HAWP02500) on a TSA plate (BD BBL) and incubated at 37°C for 24 h. After incubation, filters were removed using sterile forceps, and the bacteria were resuspended in 1.5 mL of sterile LB before serial dilution in LB and plating onto PIA and SIA. After incubation at 37°C overnight, colonies were counted and the CFU per mL was calculated. The fold change of S. aureus CFU/mL was calculated by dividing the CFU/mL of S. aureus (either CF isolate or JE2 control) grown with P. aeruginosa (either CF isolate or nonmucoid/mucoid PAO1) over the CFU/mL of each S. aureus isolate grown in monoculture (Fig. S1). The fold change of P. aeruginosa CFU/mL was calculated by dividing the CFU/mL of P. aeruginosa (either CF isolate or nonmucoid/mucoid PAO1 control) grown with S. aureus (either CF isolate or JE2 control) over the CFU/mL of each P. aeruginosa isolate grown in monoculture. All coculture experiments were performed in technical duplicates and at least three biological replicates. The average CFU/mL for each biological replicate was calculated from the two technical replicates, and this average was used to calculate the CFU/mL fold change for each biological replicate. The average CFU/mL fold change was calculated across all biological replicates for each coculture group, and these data are represented in boxplots. To ensure consistency, S. aureus JE2 paired with PAO1 (both mucoid and nonmucoid) was included as a control in each assay. We observed a JE2 CFU/mL fold change of ~10^−1^ when it was cocultured with mucoid PAO1 and ~10^−3^ to 10^−4^ when it was cocultured with nonmucoid PAO1 with high reproducibility.

### Statistical analysis.

The CFU/mL fold change values for the groups of coinfection pairs were tested for normality using the Shapiro-Wilk test. *P* values of <0.05 were considered nonnormal distributions. The CFU/mL fold change values were then statistically compared using Welch’s *t* test or the Wilcoxon rank sum test depending on whether or not the data were normally distributed, and *P* values of <0.05 were considered statistically significant. Statistical tests were performed using the shapiro.test, t.test, and wilcox.test functions in R. Welch’s *t* test with false-discovery rate correction was used to compare all individual coinfection pairs using the pairwise_t_test function from the rstatix package (Table S2).

### Data availability.

The P. aeruginosa coinfection isolates have also been sequenced; the draft assemblies and the raw Illumina reads have been deposited in NCBI and are available under BioProject accession number PRJNA776003.
