# Supplementary material for: Staphylococcus aureus and Pseudomonas aeruginosa Isolates from the Same Cystic Fibrosis Respiratory Sample Coexist in Coculture
Source: Microbiol Spectr. 2022 Jul 18;10(4):e00976-22. doi: 10.1128/spectrum.00976-22 (PMC9431432; doi:10.1128/spectrum.00976-22)
Supplement: Supplemental file 1 — Supplemental material. Download spectrum.00976-22-s0001.pdf, PDF file, 1.1 MB [file spectrum.00976-22-s0001.pdf]

Supplementary Material

Staphylococcus aureus and *Pseudomonas aeruginosa* isolates from the same cystic fibrosis respiratory sample coexist in coculture

Eryn E. Bernardy[1,2#], Vishnu Raghuram[2,3#], and Joanna B. Goldberg[2*]

[1]Department of Biology, Elon University, Elon, North Carolina, USA

[2]Division of Pulmonary, Asthma, Cystic Fibrosis, and Sleep, Department of Pediatrics, Emory University School of Medicine, Atlanta, Georgia, USA

[3]Microbiology and Molecular Genetics Program, Graduate Division of Biological and Biomedical Sciences, Laney Graduate School, Emory University, Atlanta, Georgia, USA

[#]Authors contributed equally. The order was determined by seniority.

[*]Corresponding author: joanna.goldberg@emory.edu

Running title: Concurrent *Staphylococcus* and *Pseudomonas* coexist

Table S1: Summary of raw coculture data obtained from cystic fibrosis (CF) *S. aureus* (Sa) and CF *P. aeruginosa* (Pa) obtained from cystic fibrosis patients. Patient ID number is indicated along with date of sample collection and type of sample. *S. aureus* CFU/mL fold change and *P. aeruginosa* CFU/mL fold change was determined as described in Materials and Methods. For "Clinical Sa vs Mucoid/Nonmucoid PAO1*", this was previously reported in Bernardy et al. 2020 (1); values were included for mucoid PAO1 when the CF *P. aeruginosa* was mucoid and for nonmucoid PAO1 when the CF *P. aeruginosa* was nonmucoid, otherwise #. "NA" = not available.

Table S2: Individual comparisons of CFU/mL fold change of each cystic fibrosis (CF) *S. aureus* (Sa) and *P. aeruginosa* (Pa) isolate when competed against the corresponding co-infection isolate or laboratory isolate. Comparisons were done using pairwise Welch's t tests with False Discovery Rate (FDR) correction. Columns 1, 2 and 3 show the co-infection pair (Sa vs. Pa), the patient ID, and the mucoidy status of the co-infecting *P. aeruginosa*, respectively. Columns 4 and 5 show the two groups whose CFU/mL fold changes are being compared. The number of biological replicates in each group is shown in columns 6 and 7. Columns 8 and 9 show the Welch's p and FDR adjusted p value. Column 10 shows the significance of the FDR p (* = significant, ns = not significant)

Table S3: CFU/mL fold changes of cystic fibrosis (CF) *S. aureus* (Sa) isolates when cocultured with non-co-infecting CF *P. aeruginosa* (Pa) isolates. Each row shows the CFU/mL fold change of a *S. aureus* isolate when cocultured with its corresponding co-infection *P. aeruginosa* isolate (Clinical Sa vs. Co-infecting Pa) and with an alternate non-co-infecting *P. aeruginosa* isolate (Clinical Sa vs. Non-co-infecting Pa). Three *P. aeruginosa* isolates were chosen for the non-co-infection cocultures (CFBR309_Pae_20170510_S_EBPa20, CFBR120_Pae_20120627_S_Pa41, and CFBR123_Pae_20120222_S_Pa43). These data are also represented in **Figure S3**.

Figure S1: CFU/mL fold change of cystic fibrosis (CF) *S. aureus* (Sa) /JE2 when cocultured with CF *P. aeruginosa* (Pa)/PAO1. Each row represents *S. aureus* and *P. aeruginosa* strains isolated from a single patient. Bars on each box represent mean CFU/mL fold change of *S. aureus*. In each box, left bar represents CF *S. aureus* cocultured with its concurrently isolated CF *P. aeruginosa* strain. Middle bar represents the same CF *S. aureus* strain cocultured with mucoid (black) or non-mucoid (green) lab strain PAO1, which corresponds to the mucoidy of the CF *P. aeruginosa* strain. Right bar represents reference strain JE2 cocultured with the CF isolate of *P. aeruginosa* that corresponds to the patient. Boxes drawn side-by-side represent multiple isolates obtained from the same patient. Boxes connected by a red line show the same *S. aureus* strain isolated concurrently with multiple *P. aeruginosa* strains from the same patient. Boxes not connected by a red line show different *S. aureus* and *P. aeruginosa* strains isolated from the same patient over time. Key figure at bottom of page shows x and y axes labels, box headers and legends.

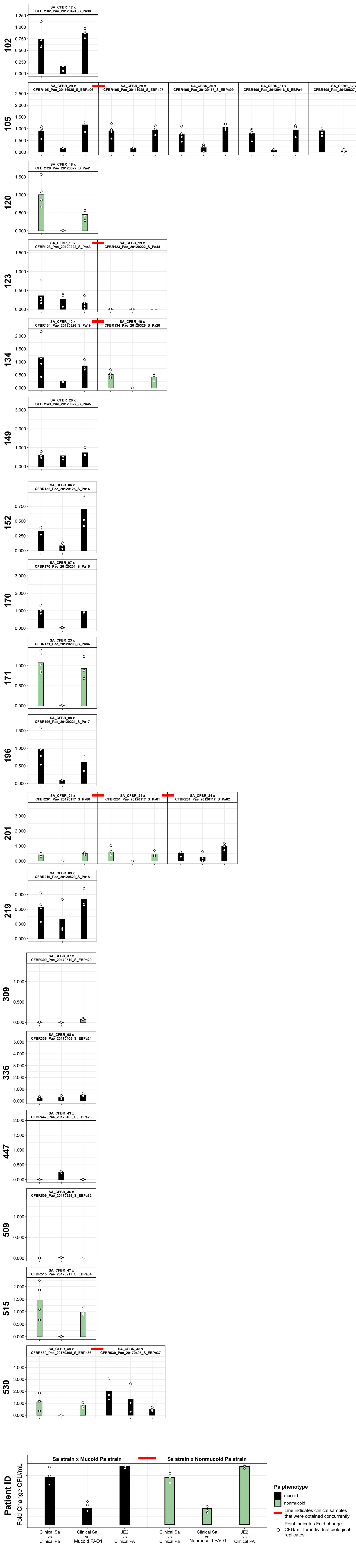

102
SA_CFBR_17 x CFBR102_Pae_20120424_S_Pa38
105
SA_CFBR_29 x CFBR105_Pae_20111025_S_EBPa06
SA_CFBR_29 x CFBR105_Pae_20111025_S_EBPa07
SA_CFBR_30 x CFBR105_Pae_20120117_S_EBPa09
SA_CFBR_31 x CFBR105_Pae_20120416_S_EBPa11
SA_CFBR_32 x CFBR105_Pae_20120627_S_EBPa13
SA_CFBR_33 x CFBR105_Pae_20120802_S_EBPa15
120
SA_CFBR_18 x CFBR120_Pae_20120627_S_Pa41
123
SA_CFBR_19 x CFBR123_Pae_20120222_S_Pa43
SA_CFBR_19 x CFBR123_Pae_20120222_S_Pa44
134
SA_CFBR_10 x CFBR134_Pae_20120326_S_Pa19
SA_CFBR_10 x CFBR134_Pae_20120326_S_Pa20
149
SA_CFBR_20 x CFBR149_Pae_20120627_S_Pa45
152
SA_CFBR_06 x CFBR152_Pae_20120125_S_Pa14
170
SA_CFBR_07 x CFBR170_Pae_20120201_S_Pa15
171
SA_CFBR_23 x CFBR171_Pae_20120208_S_Pa84
196
SA_CFBR_08 x CFBR196_Pae_20120221_S_Pa17
201
SA_CFBR_24 x CFBR201_Pae_20120117_S_Pa80
SA_CFBR_24 x CFBR201_Pae_20120117_S_Pa81
SA_CFBR_24 x CFBR201_Pae_20120117_S_Pa82
219
SA_CFBR_09 x CFBR219_Pae_20120529_S_Pa18
309
SA_CFBR_37 x CFBR309_Pae_20170510_S_EBPa20
336
SA_CFBR_08 x CFBR336_Pae_20170405_S_EBPa24
447
SA_CFBR_43 x CFBR447_Pae_20170405_S_EBPa28
509
SA_CFBR_46 x CFBR509_Pae_20170525_S_EBPa32
515
SA_CFBR_47 x CFBR515_Pae_20170217_S_EBPa34
530
SA_CFBR_48 x CFBR530_Pae_20170405_S_EBPa36
SA_CFBR_48 x CFBR530_Pae_20170405_S_EBPa37
Patient ID
Fold Change CFU/mL
Sa strain x Mucoid Pa strain
Sa strain x Nonmucoid Pa strain
Clinical Sa vs Clinical Pa
Clinical Sa vs Mucoid PAO1
JE2 vs Clinical PA
Clinical Sa vs Clinical Pa
Clinical Sa vs Nonmucoid PAO1
JE2 vs Clinical PA
Pa phenotype
mucoid
nonmucoid
Line indicates clinical samples that were obtained concurrently
Point indicates Fold change CFU/mL for individual biological replicates

Figure S2: Cystic fibrosis (CF) *S. aureus* can either survive or be killed by CF *P. aeruginosa* (Pa) from different patients (non-co-infecting isolates). Three *P. aeruginosa* isolates (**Panel A:** CFBR309_Pae_20170510_S_EBPa20; **Panel B:** CFBR120_Pae_20120627_S_Pa41; and **Panel C:** CFBR123_Pae_20120222_S_Pa43) were each competed against four *S. aureus* from different patients. Each box represents one of the three *P. aeruginosa* isolates (Box title). Dots in the left column represent average CFU/mL fold change of the *S. aureus* isolate that was co-infecting with the representative *P. aeruginosa*, same data as shown in **Figure 1**. Dots in the middle column represent CFU/mL fold changes of four different *S. aureus* isolates that were not co-infecting with the representative *P. aeruginosa*. Dots in the right column represent the CFU/mL fold changes of the same four *S. aureus* isolates as in the middle column but when competed against their respective co-infecting *P. aeruginosa* isolates, same data as shown in **Figure 1**. Dashed lines connect the same *P. aeruginosa* isolate (left → middle) or the same *S. aureus* isolates (middle → right). Raw data for this figure are shown in **Table S3**.

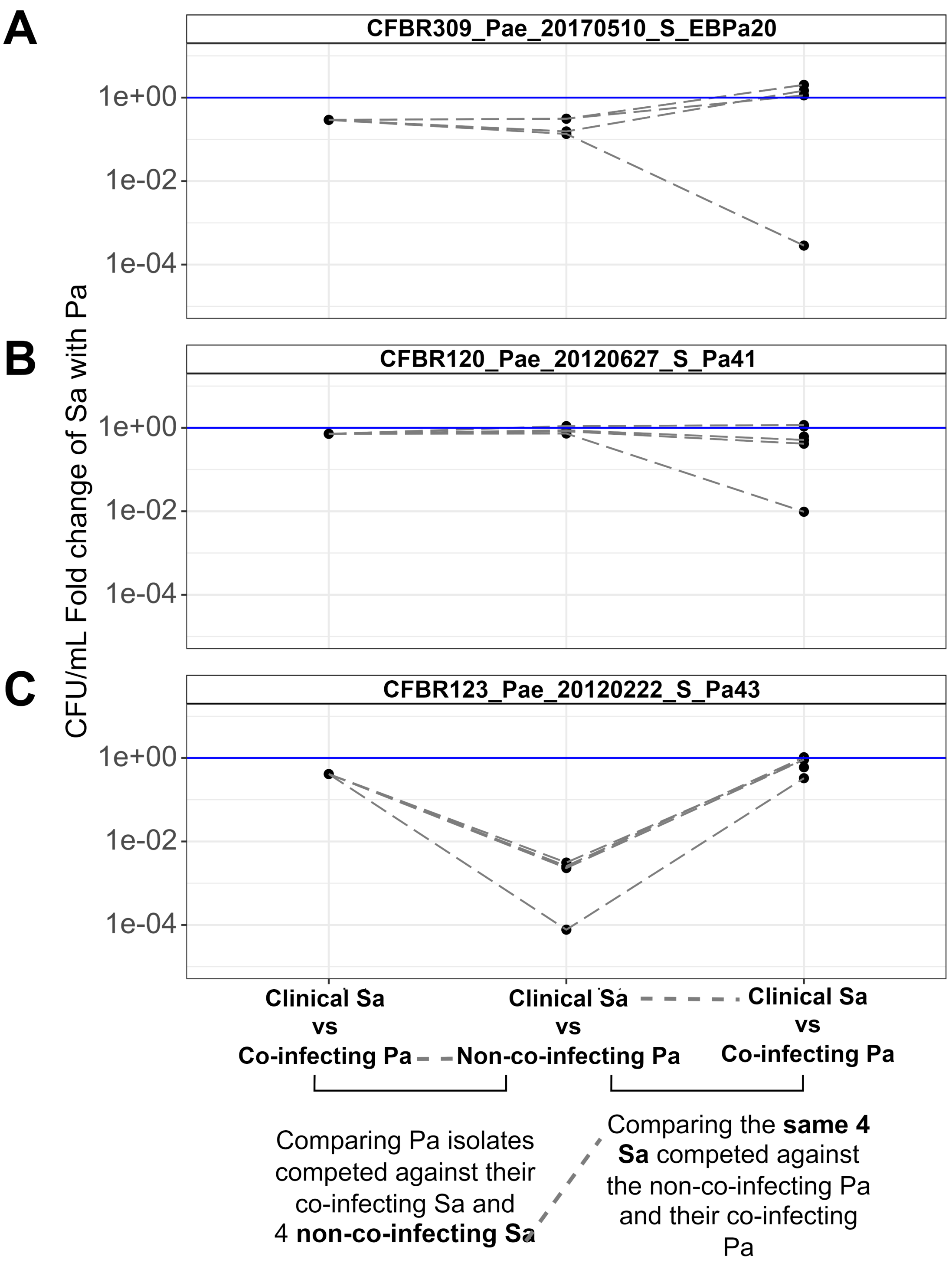
A
CFBR309_Pae_20170510_S_EBPa20
B
CFBR120_Pae_20120627_S_Pa41
C
CFBR123_Pae_20120222_S_Pa43
CFU/mL Fold change of Sa with Pa
1e+00
1e-02
1e-04
Clinical Sa vs Co-infecting Pa
Clinical Sa vs Non-co-infecting Pa
Clinical Sa vs Co-infecting Pa
Comparing Pa isolates competed against their co-infecting Sa and 4 non-co-infecting Sa
Comparing the same 4 Sa competed against the non-co-infecting Pa and their co-infecting Pa

References:

1. Bernardy EE, Petit RA, 3rd, Raghuram V, Alexander AM, Read TD, Goldberg JB. Genotypic and phenotypic diversity of *Staphylococcus* aureus iolates from cystic fibrosis patient lung infections and their interactions with *Pseudomonas aeruginosa*. mBio. 2020;11(3). Epub 2020/06/25. doi: 10.1128/mBio.00735-20. PubMed PMID: 32576671; PMCID: PMC7315118.
